# Supplementary material for: MicroRNAs Signature Panel Identifies Heavy Drinkers with Alcohol-Associated Cirrhosis from Heavy Drinkers without Liver Injury
Source: Biology (Basel). 2023 Oct 8;12(10):1314. doi: 10.3390/biology12101314 (PMC10604848; doi:10.3390/biology12101314)
Supplement: Supplementary file 1 [file biology-12-01314-s001.zip › biology-2610653-supplementary.pdf]

Supplementary Materials: MicroRNAs Signature Panel Identifies Heavy Drinkers with Alcohol-Associated Cirrhosis from Heavy Drinkers without Liver Injury

Fathima Shihana, Mugdha V. Joglekar, Tae-Hwi Schwantes-An, Anandwardhan A. Hardikar and Devanshi Seth

Table S1: List of microRNAs resulting from different analyses

Cases vs drinking controls	Correlation analysis of 21 miRNA expression (rCt) with traditional biomarkers	miRNAs by best performance AUC-ROC	AH vs Cirrhosis
miR-16	miR-16	miR-191	miR-15a
miR-19a	miR-19a	miR-27a	**miR-21-5p**
miR-19b	miR-27a	miR-130a	**miR-28**
miR-26a	miR-29b		miR-125b-5p
miR-27a	miR-101		and miR-185
miR-27b	miR-130a		
miR-29b	miR-191		
miR-30c			
miR-101			
miR-130a			
miR-151-3p			
miR-152			
miR-191			
miR-199a-3p			
miR-221			

miR-301
miR-335
miR-379
miR-374-5p
miR-652
miR-532-3p

Upregulated micoRNAs are in bold. Controls are drinking controls with no liver disease; Cases are drinkers with cirrhosis and alcoholic hepatitis; Cirrhosis are drinkers with cirrhosis (no AH); AH are drinkers with alcoholic hepatitis.

Table S2: Correlation analysis of miRNA expression (△Ct Ct) with traditional biomarkers.

miRs	Albumin(umol/l)	Platelet count (10^9/L)	GGT(IU/L)	BMI	Haemoglobin (g/L)
miR-16	-0.31* (-0.55 to -0.01)	-0.38* (-0.59 to 0.02)	-0.01 (-0.29 to 0.29)	0.19 (-0.11 to 0.45)	-0.24 (-0.50 to 0.05)
miR-19a	-0.31* (-0.55 to -0.01)	-0.34* (-0.58 to -0.05)	0.19 (-0.10 to 0.46)	0.22 (-0.07 to 0.49)	-0.17 (-0.44 to 0.12)
miR-19b	-0.16 (-0.43 to 0.14)	-0.29* (-0.54 to 0.01)	0.12 (-0.17 to 0.40)	0.14 (-0.16 to 0.41)	-0.24 (-0.49 to 0.06)
miR-26a	-0.16 (-0.43 to 0.13)	-0.40** (-0.62 to -0.12)	0.34* (0.05 to 0.58)	0.24 (-0.05 to 0.50)	-0.25 (-0.51 to 0.04)
miR-27a	-0.05 (-0.33 to 0.24)	-0.30* (-0.54 to -0.01)	0.22 (-0.07 to 0.48)	0.34* (0.05 to 0.57)	-0.13 (-0.41 to 0.16)
miR-27b	-0.25 (-0.51 to 0.04)	-0.24 (-0.50 to 0.05)	0.11 (-0.18 to 0.40)	0.06 (-0.23 to 0.34)	-0.18 (-0.45 to 0.12)
miR-29c	-0.29* (-0.54 to -0.01)	-0.25 (-0.51 to 0.04)	0.31 (0.02 to 0.55)	0.28 (0.01 to 0.53)	-0.28 (-0.53 to 0.01)
miR-30c	-0.22 (-0.48 to 0.07)	-0.41** (-0.63 to -0.13)	0.05 (-0.23 to 0.34)	0.26 (-0.03 to 0.52)	-0.32* (-0.56 to -0.02)
miR-101	-0.20 (-0.46 to 0.09)	-0.29* (-0.53 to 0.02)	0.33* (0.04to 0.57)	0.26 (-0.03 to 0.52)	-0.19 (-0.46 to 0.10)
miR-130a	-0.30* (-0.55 to -0.01)	-0.40**(-0.62 to -0.11)	0.26 (-0.03 to 0.52)	0.22 (-0.07 to 0.48)	-0.40** (-0.62 to -0.11)
miR-151-3p	-0.19 (-0.46 to 0.10)	-0.35*(-0.58 to -0.06)	-0.01 (-0.30 to 0.28)	0.31* (0.02 to 0.55)	-0.25 (-0.51 to 0.04)
miR-191	-0.30*(-0.55 to -0.01)	-0.44** (-0.65 to -0.17)	0.17 (-0.13 to 0.44)	0.30* (0.01 to 0.55)	-0.10 (-0.38 to 0.20)
miR-199a-3p	-0.06 (-0.35 to 0.23)	-0.31*(-0.55 to -0.01)	0.12 (-0.17 to 0.40)	0.25 (-0.04 to 0.51)	-0.23 (-0.54 to 0.13)
miR-221	-0.21 (-0.47 to 0.08)	-0.34* (-0.57 to -0.05)	0.34* (0.05 to 0.57)	0.20 (-0.09 to 0.46)	-0.24 (-0.50 to 0.05)

miR-335	-0.16 (-0.43 to 0.14)	-0.36* (-0.59 to -0.07)	0.31* (0.02 to 0.55)	0.32* (0.02 to 0.56)	-0.31* (-0.55 to -0.02)
miR-374-5p	-0.22 (-0.48 to 0.07)	-0.20 (-0.46 to 0.10)	0.27 (-0.02to 0.52)	0.20 (-0.09 to 0.47)	-0.27 (-0.52 to 0.02)
miR-532-3p	-0.16 (-0.44 to 0.13	-0.24 (-0.49 to 0.05)	0.07 (-0.22 to 0.35)	0.18 (-0.11 to 0.45)	-0.17 (-0.44 to 0.13)
miR-652	-0.28 (-0.53 to 0.01)	-0.36*(-0.59 to -0.07)	0.20 (-0.10 to 0.46)	0.18 (-0.11 to 0.45)	-0.14 (-0.42 to 0.15)

Table S3: Correlation of miRNA expression with genetic risk variants for alcohol-associated cirrhosis

	PNPLA3 **rs2294915**	*TM6SF2* **rs10401969**	*HSD17B13* **rs10433937**	*FAF2* **rs11134977**	*SERPINA1* **rs28929474**	*MARC1* **rs2642438**	*MBOAT7* **rs641738**
miR-16	0.06	0.04	0.25	0.13	-0.03	-0.06	-0.3
miR-19a	0.05	0.04	**0.51*****	-0.04	-0.09	-0.08	0
miR-19b	0.11	0.03	0.22	0.12	0	-0.03	-0.04
miR-26a	-0.13	0.26	**0.32***	-0.01	0.05	-0.11	0
miR-27a	-0.03	0.19	0.2	0.3	-0.04	-0.19	0.11
miR-27b	0.25	**0.33***	0.17	-0.01	0	-0.2	-0.04
miR-29c	0.01	0.23	0.06	0.29	0.05	0.01	0.22
miR-30c	0.09	0.21	0.19	0.13	0.12	-0.02	-0.13
miR-101	0.09	0.28	**0.32***	0.14	0.11	0.08	-0.1
miR-130a	0.04	0.2	0.22	**0.34***	0.22	0.02	0.19
miR-151-3p	-0.04	0.17	**0.38***	0.18	0.15	0.03	-0.02
miR-152	0.2	-0.17	0.3	0.1	0.14	0.2	-0.05
miR-191	0.11	0.13	0.3	0.13	0.23	0.04	-0.09
miR-199a-3p	0.05	0.19	0.3	-0.03	0.07	-0.28	0.19
miR-221	0.08	0.2	**0.32***	0.1	0.08	-0.21	0.19
miR-301	-0.12	0.19	**0.45****	0.1	0.14	-0.07	-0.29
miR-335	0.31	-0.08	0.28	-0.15	0.09	**-0.35***	0.03

miR-374-5p	-0.26	0.23	0.07	0.23	0.09	0.27	0.17
miR-379	0.09	0.17	0.16	0.13	0.07	-0.22	0.11
miR-410	-0.2	0	0.08	0.26	0.07	-0.14	-0.03
miR-532-3p	0.1	0.28	0.18	0.21	0.11	-0.07	-0.08
miR-652	0.08	-0.01	0.02	0.04	0.09	-0.19	0.15

Significant correlation coefficients are bold

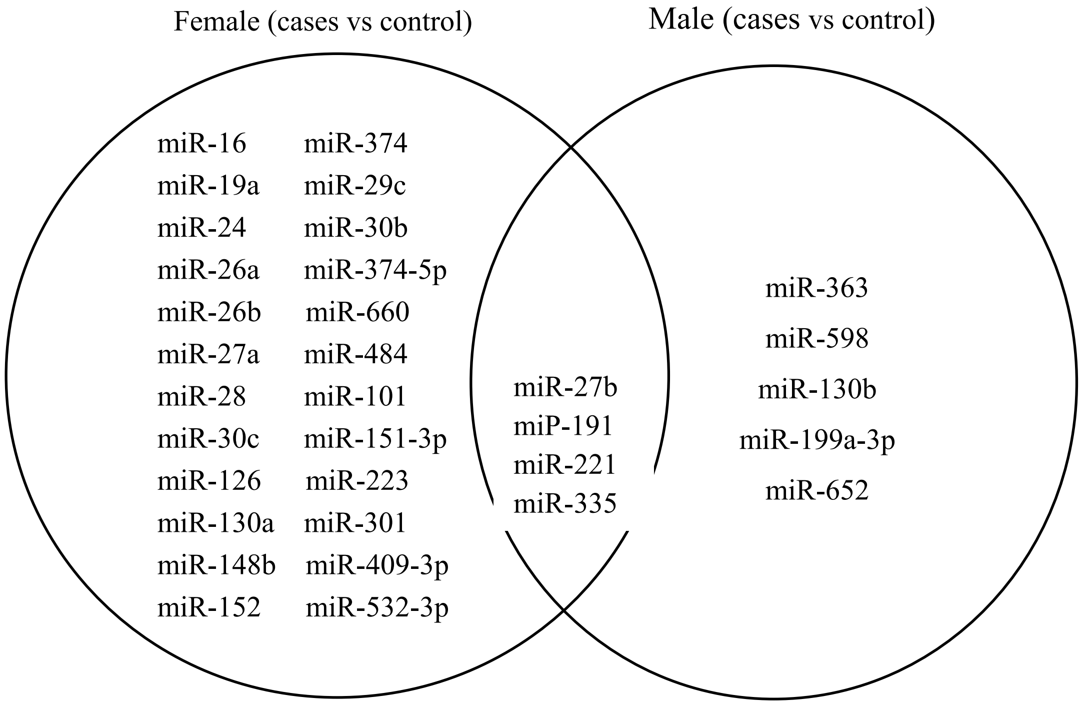

Figure S1: Differentially expressed microRNAs in male and female cases compared to drinking controls. Venn diagram shows significantly downregulated microRNAs in cases versus drinking controls by female/male gender. Four microRNAs are commonly downregulated in both female and male cases.

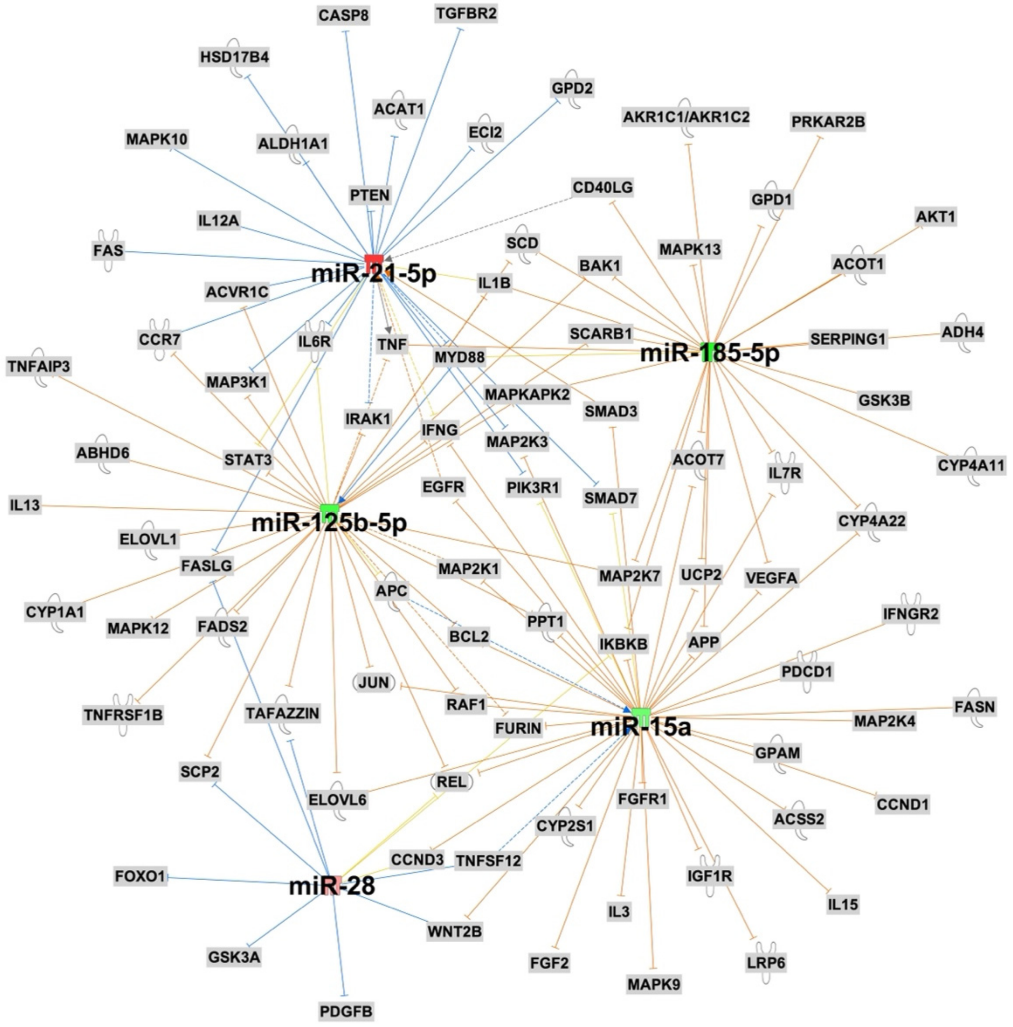

© 2000-2022 QIAGEN. All rights reserved.

a)

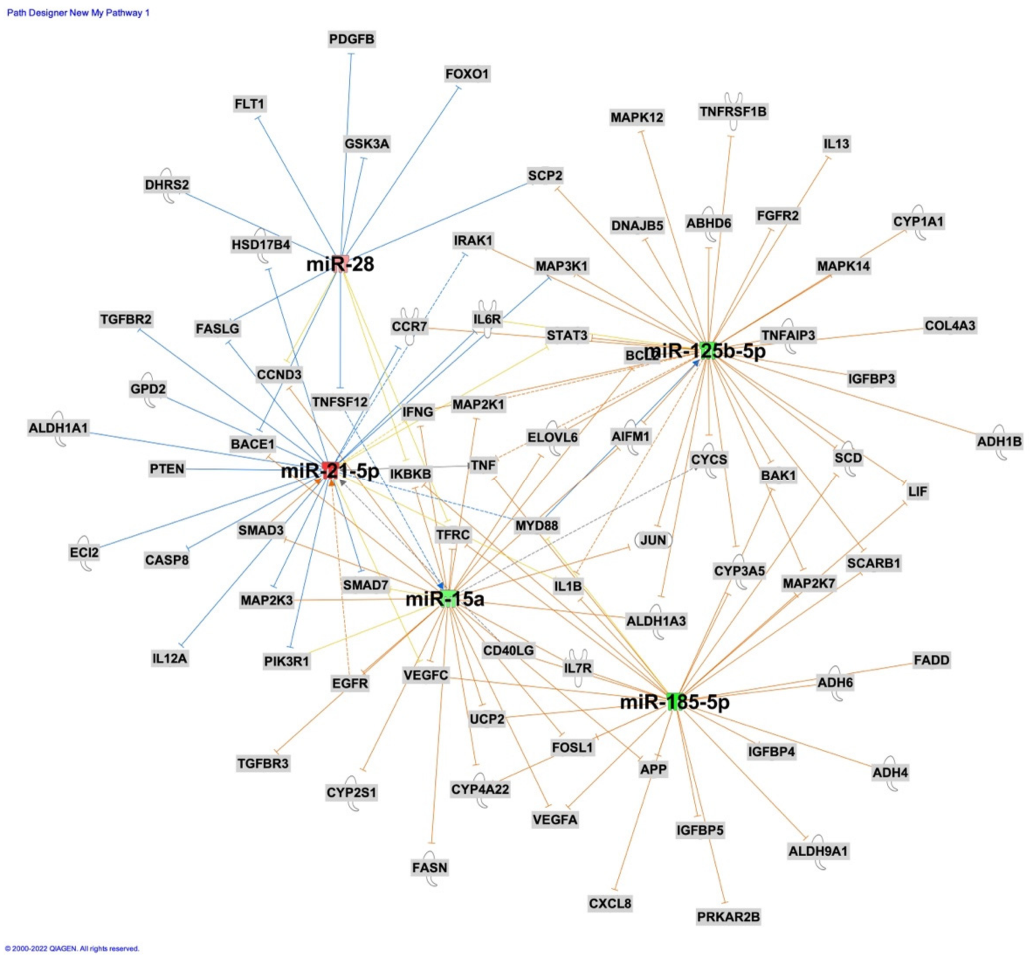

b)

Figure S2: Target mRNA and microRNA-mRNA interactions derived from select AH specific microRNAs. Target mRNAs of 5 microRNAs associated with AH were overlaid on **a)** disease and functions, and **b)** Toxic functions using 'Pathway design' option in IPA. The top interaction networks are presented.
